# Shotgun Metagenomics of Gut Microbiota in Humans with up to Extreme Longevity and the Increasing Role of Xenobiotic Degradation

**DOI:** 10.1128/mSystems.00124-20

**Published:** 2020-03-24

**Authors:** Simone Rampelli, Matteo Soverini, Federica D’Amico, Monica Barone, Teresa Tavella, Daniela Monti, Miriam Capri, Annalisa Astolfi, Patrizia Brigidi, Elena Biagi, Claudio Franceschi, Silvia Turroni, Marco Candela

**Affiliations:** a Unit of Microbial Ecology of Health, Department of Pharmacy and Biotechnology, University of Bologna, Bologna, Italy; b Department of Experimental and Clinical Biomedical Sciences Mario Serio, University of Florence, Florence, Italy; c Department of Experimental, Diagnostic and Specialty Medicine, University of Bologna, Bologna, Italy; d CIG–Interdepartmental Center Galvani, University of Bologna, Bologna, Italy; e CSR–Centro di Studio per la Ricerca dell’Invecchiamento, University of Bologna, Bologna, Italy; f Giorgio Prodi Cancer Research Center, University of Bologna, Bologna, Italy; g Interdepartmental Centre of Industrial Agrifood Research (CIRI-Agrifood), University of Bologna, Cesena, Italy; h IRCCS, Institute of Neurologic Sciences of Bologna, Bellaria Hospital, Bologna, Italy; i Department of Applied Mathematics, Institute of Information Technology, Lobachevsky University of Nizhny Novgorod, Nizhny Novgorod, Russia; Duke University

**Keywords:** microbiome, metagenome, extreme longevity, xenobiotics, aging

## Abstract

The gut microbiome of long-lived people display an increasing abundance of subdominant species, as well as a rearrangement in health-associated bacteria, but less is known about microbiome functions. In order to disentangle the contribution of the gut microbiome to the complex trait of human longevity, we here describe the metagenomic change of the human gut microbiome along with aging in subjects with up to extreme longevity, including centenarians (aged 99 to 104 years) and semisupercentenarians (aged 105 to 109 years), i.e., demographically very uncommon subjects who reach the extreme limit of the human life span. According to our findings, the gut microbiome of centenarians and semisupercentenarians is more suited for xenobiotic degradation and shows a rearrangement in metabolic pathways related to carbohydrate, amino acid, and lipid metabolism. Collectively, our data go beyond the relationship between intestinal bacteria and physiological changes that occur with aging by detailing the shifts in the potential metagenomic functions of the gut microbiome of centenarians and semisupercentenarians as a response to progressive dietary and lifestyle modifications.

**IMPORTANCE** The study of longevity may help us understand how human beings can delay or survive the most frequent age-related diseases and morbidities. In this scenario, the gut microbiome has been proposed as one of the variables to monitor and possibly support healthy aging. Indeed, the disruption of host-gut microbiome homeostasis has been associated with inflammation and intestinal permeability as well as a general decline in bone and cognitive health. Here, we performed a metagenomic assessment of fecal samples from semisupercentenarians, i.e., 105 to 109 years old, in comparison to young adults, the elderly, and centenarians, shedding light on the longest compositional and functional trajectory of the human gut microbiome with aging. In addition to providing a fine taxonomic resolution down to the species level, our study emphasizes the progressive age-related increase in degradation pathways of pervasive xenobiotics in Western societies, possibly as a result of a supportive process within the molecular continuum characterizing aging.

## Abstract (precis)

The study of longevity may help us understand how human beings can delay or survive the most frequent age-related diseases and morbidities. In this scenario, the gut microbiome has been proposed as one of the variables to monitor and possibly support healthy aging. Indeed, the disruption of host-gut microbiome homeostasis has been associated with inflammation and intestinal permeability as well as a general decline in bone and cognitive health. Here, we performed a metagenomic assessment of fecal samples from semisupercentenarians, i.e., 105 to 109 years old, in comparison to young adults, the elderly, and centenarians, shedding light on the longest compositional and functional trajectory of the human gut microbiome with aging. In addition to providing a fine taxonomic resolution down to the species level, our study emphasizes the progressive age-related increase in degradation pathways of pervasive xenobiotics in Western societies, possibly as a result of a supportive process within the molecular continuum characterizing aging.

## INTRODUCTION

Longevity has been described as the result of a complex combination of variables, deriving from genetics, lifestyle, and environment (1, 2). In this context, the intestinal microbiome has been proposed as a possible mediator of healthy aging that preserves host-environment homeostasis by counteracting inflammaging (3, 4), intestinal permeability (5), and deterioration of cognitive and bone health (5, 6). Correlations have been previously found between age-related gut microbiota dysbioses and levels of proinflammatory cytokines, hospitalization, poor diet, and frailty in the elderly (7). More recently, the longest human gut microbiota trajectory with aging has been built by comparing the fecal bacterial taxa from healthy adults and older individuals, including semisupercentenarians, i.e., people aged 105 to 109 years (8, 9). However, the functional changes that occur in the gut microbiome along with aging are still largely unexplored. In an attempt to provide some glimpses in this direction and to advance our knowledge on whether and how the gut microbiome may support the maintenance of health in extreme aging, we here characterized the fecal microbiome of 62 individuals, with ages ranging from 22 to 109 years, by shotgun metagenomics. According to our findings, aging is characterized by an increased number of genes involved in xenobiotic degradation, as well as by rearrangements in metabolic pathways related to carbohydrate, amino acid, and lipid metabolism. These microbiome features are boosted even more in semisupercentenarians, probably representing the result of a lifelong remodeling response to progressive changes in diet and lifestyle.

## RESULTS

We previously found considerable age-related variability in fecal microbiota composition of 69 people, including centenarians and semisupercentenarians, from the Emilia Romagna region of Italy and the surrounding area (8). In an attempt to go further, unraveling the functional and species-level taxonomic links between the gut microbiome and extreme aging, we applied shotgun metagenomics to a subset of 62 DNA samples derived from the same data set previously analyzed (8). Specifically, we characterized the gut microbiome from 11 young adults (group Y, 6 females and 5 males, aged 22 to 48 years [mean age, 32.2 years]), 13 younger elderly (group K, 6 females and 7 males, aged 65 to 75 years [mean age, 72.5 years]), 15 centenarians (group C, 14 females and 1 male, aged 99 to 104 years [mean age, 100.4 years]), and 23 semisupercentenarians (group S, 17 females and 6 males, aged 105 to 109 years [mean age, 106.3 years]). A total of 1.3 billion sequences were generated, with an average of 20 million reads (±5 million reads standard deviation [SD]) per subject.

**FIG 1:**
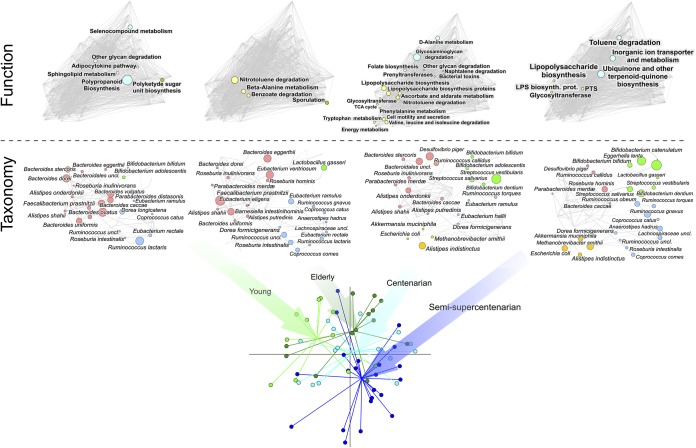
Gut microbiome variation with aging. (Top) Network plots showing the taxonomic and functional configurations of the gut microbiome of four age groups: 11 young adults (aged 22 to 48 years; group young), 13 younger elderly (aged 65 to 75 years; group elderly), 15 centenarians (aged 99 to 104 years; group centenarian), and 23 semisupercentenarians (aged 105 to 109 years; group semisupercentenarian). Disc sizes indicate species or functional pathway overabundances relative to the average abundance of the whole cohort. Lines indicate significant positive correlations between the values of the discs. (Bottom) PCoA plot of Bray-Curtis dissimilarity between the species-level relative abundance data sets of the four age groups.

**FIG 2:**
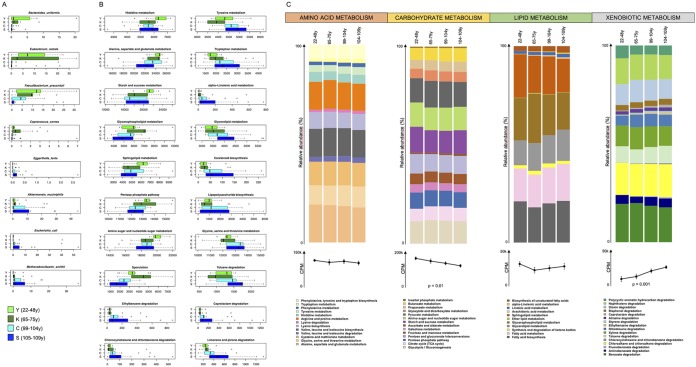
Aging-related trajectories of gut microbiome species and functional pathways. (A) Box plots of the normalized relative abundances of bacterial species differentially represented among the four age groups (Y, young adults; K, younger elderly; C, centenarians; S, semisupercentenarians) (*P* < 0.05, Kruskal-Wallis test). (B) Box plots of the normalized abundance (assigned reads per million sequences, i.e., counts per million [CPM]) of KEGG pathways differentially represented among age groups (*P* < 0.05, Kruskal-Wallis test). (C) Bar plots at the top show the KEGG pathway-classified metabolic configurations for amino acid, carbohydrate, lipid, and xenobiotic metabolism as the mean relative contribution of each pathway to the total normalized number of reads assigned to each specific metabolism. At the bottom of the panel, the average number of normalized reads (CPM ± standard error of the mean [SEM error bar]) assigned to each specific metabolism is shown. Significant differences among age groups are shown on the graphs.

We first confirmed that the fecal microbiota in all age groups is dominated by a few bacterial families (i.e., *Bifidobacteriaceae*, *Bacteroidaceae*, *Lachnospiraceae*, and *Ruminococcaceae*) whose relative abundance decreases with age (mean relative abundance ± SD: group Y, 73% ± 3%; group K, 65% ± 4%; group C, 62% ± 4%; group S, 58% ± 6%). When focusing our attention at the species level, we found that these contributions were mainly accounted for by 13 bacterial species: Bifidobacterium adolescentis, Bifidobacterium longum, Bacteroides uniformis, Faecalibacterium prausnitzii, Ruminococcus bromii, *Subdoligranulum* sp., Anaerostipes hadrus, Blautia obeum, Ruminococcus torques, Coprococcus catus, Coprococcus comes, Dorea longicatena, and *Roseburia* sp. Bray-Curtis principal-coordinate analysis (PCoA) of species-level relative abundance profiles provided evidence of an age-related trajectory (*P* < 0.05, permutation test with pseudo-F ratios), involving the establishment of age group-specific topological patterns in the taxonomic and functional microbiome structure, as shown by network plots (Fig. 1) and bar plots (see Fig. S1 in the supplemental material). However, the species-level compositional structure of the gut microbiota from the younger elderly group overall matches that from young adults (*P* = 0.2), suggesting that the physiology of the aging process may not involve gross changes in gut microbiome species and their relative abundance. On the other hand, gut microbiota from centenarians and semisupercentenarians feature a distinctive rearrangement in their taxonomic configurations (Fig. 2A). In particular, compared with younger individuals, long-lived people show a decreased contribution of B. uniformis, Eubacterium rectale, *C. comes*, and *F. prausnitzii*, along with a progressive increase of Escherichia coli, Methanobrevibacter smithii, Akkermansia muciniphila, and Eggerthella lenta (*P* < 0.05, Kruskal-Wallis test). These trends have already been reported in previous 16S rRNA gene-based microbiome works in the same subjects (3, 8), as well as in Chinese centenarians (10), further strengthening that the observed gut microbiome variations may be part of the extreme aging process, regardless of environmental variables, such as geographical origin and cultural habits (i.e., diet and lifestyle) (11).

10.1128/mSystems.00124-20.1 FIG S1 Taxonomic and functional structures of the human gut microbiome along with aging. Bar plots show the taxonomic (species level) (A) and functional (KEGG pathway level) (B) configurations of the gut microbiome of the four age groups (Y, young adults; K, younger elderly; C, centenarians; S, semisupercentenarians). Download FIG S1, PDF file, 0.7 MB. Copyright © 2020 Rampelli et al. 2020 Rampelli et al. This content is distributed under the terms of the Creative Commons Attribution 4.0 International license.

Interestingly, when we focused our analysis at a functional scale, we found a progressive age-related increase in the number of reads for genes devoted to xenobiotic biodegradation and metabolism, and a simultaneous decrease in genes involved in carbohydrate metabolism (Fig. 2B and C; Fig. S2). This functional rearrangement is even more pronounced in the gut microbiome of centenarians and semisupercentenarians, where we observed a reduced contribution of pathways for starch and sucrose (KEGG pathway no. ko00500), pentose phosphate (ko00030), and amino sugar and nucleotide sugar (ko00520) metabolism and a concomitant increase in toluene (ko00623), ethylbenzene (ko00642), caprolactam (ko00930), and chlorocyclohexane and chlorobenzene (ko00361) degradation pathways. While the changes related to carbohydrate metabolism have already been reported in previous studies and suggested to be associated with age-related changes in dietary habits (7, 9), the increase in genes for xenobiotic metabolism is reported here for the first time and appears particularly intriguing.

10.1128/mSystems.00124-20.2 FIG S2 Aging-related distribution of KEGG pathways. Box plots of the normalized abundance (assigned reads per million sequences, i.e., counts per million [CPM]) of KEGG pathways for amino acid, carbohydrate, lipid, and xenobiotic metabolism in the four age groups (Y, young adults; K, younger elderly; C, centenarians; S, semisupercentenarians). Download FIG S2, PDF file, 0.2 MB. Copyright © 2020 Rampelli et al. 2020 Rampelli et al. This content is distributed under the terms of the Creative Commons Attribution 4.0 International license.

Ethylbenzene, chlorobenzene, chlorocyclohexane, and toluene are pervasive chemicals mainly deriving from industrial manufacturing and municipal discharges and are under monitoring all over the world as part of the main environmental contaminants of the atmosphere, due to their toxic effects (12–14). The primary man-made sources of these molecules are indeed the emissions from motor and exhaust vehicles, as well as cigarette smoke. Furthermore, they are known to be generated during the processing of refined petroleum products, such as plastics, and to be contained in common consumer products, such as paints and lacquers, thinners, and rubber products (14). As regards caprolactam, it is the raw material of nylon, used for the production of many indoor products, such as synthetic fibers, resins, synthetic leather, and plasticizers. Previous studies have demonstrated the higher indoor burden of these molecules than in the outdoor environment and emphasized the exceptional importance of indoor exposure on human health (15, 16). It is a matter of fact that living in environments under strong anthropic pressures, such as the Emilia Romagna region in Italy (17, 18), results in the continuous and constant exposure to these pervasive xenobiotic substances, favoring their maintenance and progressive accumulation in body tissues, including the gut (19–22). We believe that this could create the appropriate conditions for the human host to select for gut microbiome components capable of detoxifying such chemical compounds, with a mutual benefit in terms of microbiome and host fitness in anthropic environments. Indeed, recent works have shown that the human-associated microbial communities of urban Western populations are functionally suited to the degradation of xenobiotic molecules, including caprolactam (23–25). Further supporting the importance of human microbiomes in providing a response to xenobiotic exposure, in another recent work the upper airway microbiome of nonasthmatic individuals has been found to possess greater ability to metabolize caprolactam than that of asthmatic people (25). According to the authors, the selection of caprolactam-degrading microbes in the airway microbiome would decrease host exposure to indoor air pollutants, providing an ultimate impact on human health.

**FIG 3:**
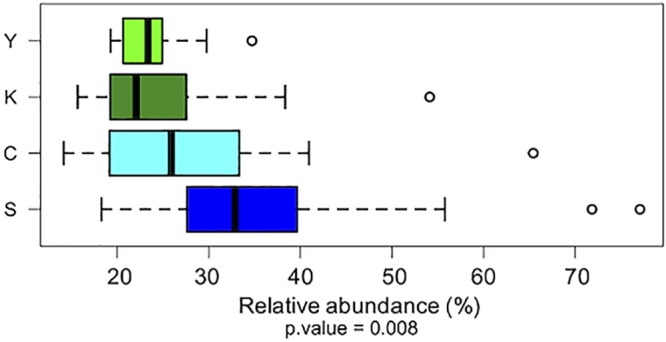
The contribution of commensal bacteria to xenobiotic degradation is significantly higher in long-lived individuals. Box plots show the percentages of bacteria of the human core gut microbiome that harbor genes for xenobiotic degradation. Members of the core microbiome were defined based on previous works (26–30).

Centenarians and semisupercentenarians are long-lived individuals who, as such, may boast an important history of exposure to xenobiotic stressors. Furthermore, as they have reduced mobility, these subjects tend to spend more time in their own houses than younger people (Fig. S3), with increased exposure to indoor pollutants. It is thus tempting to speculate that their microbiome is better equipped for the degradation of these xenobiotics as a result of a process driven by the more lasting and assiduous exposure to these chemicals. It is also worth noting that these metabolic functionalities are possessed by commensal bacteria belonging to the human core microbiome, i.e., microbial taxa that have been found to be shared by the microbiome of all human populations sampled to date (26–30) (Fig. 3). This raises important open questions on the biological mechanisms that lead to the consolidation and enrichment of xenobiotic-degrading abilities in centenarian and semisupercentenarian gut microbiomes. Here, we speculate that the highest contribution to xenobiotic degradation by commensals in long-lived people might be the result mainly of a top-down selection process related to the lifestyle habits of these exceptionally old individuals, i.e., stable and constant living settings within their own homes, together with a longer exposure and consequent accumulation of these chemicals in the host tissues due to their longer life.

10.1128/mSystems.00124-20.3 FIG S3 Centenarians and semisupercentenarians spend more time inside their own homes. Box plots show the FMOH (frequency of movement outside home) score for the subjects of groups K (younger elderly), C (centenarians), and S (semisupercentenarians). Data were not collected for individuals in group Y (young adults). “1” represents the lowest frequency and stands for “never outside the home”; “7” represents the highest frequency and stands for “daily outside the home” (different frequencies were scored from 1 up to 7 based on number of times per week, month, and year). ***, P < 0.001, Wilcoxon test. Download FIG S3, PDF file, 0.02 MB. Copyright © 2020 Rampelli et al. 2020 Rampelli et al. This content is distributed under the terms of the Creative Commons Attribution 4.0 International license.

Besides xenobiotic-degrading genes and those involved in carbohydrate metabolism, we also found age-related differences in other metabolic pathways, including those associated with lipid metabolism. In particular, centenarians and semisupercentenarians show more reads for alpha-linoleic acid (KEGG pathway no. ko00592) and glycerolipid (ko00561) metabolism; on the other hand, younger people show a greater contribution of genes involved in sphingolipid (ko00600) and glycerophospholipid (ko00564) metabolism. Given that glycerophospholipids and sphingolipids are known to be more abundant in animal-derived foods (31, 32), while alpha-linoleic acid is derived mainly from plant foods (33), these profiles may be related to eating habits and, in particular, to the higher intake of plant-derived fats than animal fats by long-lived individuals than by younger people (Fig. S4). Moreover, when looking at functional pathways involved in amino acid metabolism, we found a progressive increase with age in genes for the metabolism of tryptophan (ko00380), tyrosine (ko00350), glycine, serine, and threonine (ko00260). On the other hand, genes for alanine, aspartate, and glutamate metabolism (ko00250) were found to be more abundant in younger individuals. These evidences are in agreement with our previous study (9), in particular with regard to the metabolism of tryptophan and tyrosine as an indicator of enhanced proteolytic metabolism. Furthermore, these findings fit with metabolite measures in the centenarians of our cohort, i.e., the decrease of the bioavailability of tryptophan in serum (34), as well as the increased urinary levels of phenolic metabolites, deriving from the metabolism of tyrosine (35). Finally, we found a progressive increase with aging of genes for lipopolysaccharide biosynthesis (ko00540), which can be associated with the presence of pathobionts (i.e., members of the *Enterobacteriaceae* family) and the low levels of chronic inflammation (i.e., inflammaging), as previously demonstrated in long-lived people (3, 8, 9).

10.1128/mSystems.00124-20.4 FIG S4 Semisupercentenarians consume more vegetable fats than animal fats than young adults. Plot show the ratio between daily intake (g/day) of animal and vegetable fats for subjects of groups Y (young adults) and S (semisupercentenarians). Download FIG S4, PDF file, 0.01 MB. Copyright © 2020 Rampelli et al. 2020 Rampelli et al. This content is distributed under the terms of the Creative Commons Attribution 4.0 International license.

## DISCUSSION

Here we described—as far as we know, for the first time—the metagenomic changes of the human gut microbiota that occur with aging, up to extreme longevity, by characterizing the microbiome of semisupercentenarians, i.e., demographically very uncommon subjects who reach the extreme limit of the human life span (>105 years of age). In addition to confirming the known taxonomic features of an aging microbiota, we extended the definition of the human core gut microbiota down to the species level and provided an accurate depiction of the functional changes occurring along with aging. In a sort of continuum line with our previous study, where we demonstrated that the intestinal microbiome of Italian adults is equipped for the degradation of xenobiotics, probably as a functional response to exposure to these compounds (24), we here advance the fascinating hypothesis that aging in Western urban environments progressively selects for commensal microbiome strains with metabolic abilities toward specific xenobiotics. We speculate that this could represent an adaptive response of the human holobiont to the increased exposure to, and accumulation of, xenobiotic substances along the aging process. As recently discussed (36), future studies should be aimed at better understanding the complex interplay between xenobiotic exposure and the human gut microbiome. The individual gut microbiome structure will have to be matched with the personal exposure level, with the latter being dissected by monitoring xenobiotics in feces and body fluids. Long-term longitudinal studies must be conceived, with the aim of highlighting the mechanisms underlying this potential microbiome adaptive variation, as a result of a top-down selection process of microbiome functions for xenobiotic detoxification and the ultimate impact in terms of host health protection. Given that the xenobiotics that emerged in the present study are now ubiquitous in modern urban areas, it would also be interesting to assess the xenobiotic degradation capacity of ancient microbial communities by analyzing samples from the preindustrial era, in order to fully understand the effects of these molecules on the evolutionary history of the human holobiont. Studies of this type would help to shed light on whether the peculiar functional profiles of the gut microbiome of extremely long-lived hosts, as found in our work, are the result of an adaptive and remodeling process inherent to the physiology of human aging in modern urban societies and thus capable of supporting a new homeostasis.

## MATERIALS AND METHODS

### Subjects and study groups.

The study used genomic DNA from 62 fecal samples collected for a study by Biagi et al. (8). Subjects were enrolled in the Emilia Romagna region (Italy) and categorized as follows: 11 young adults (group Y, 6 females and 5 males, aged 22 to 48 years [mean age, 32.2 years]), 13 younger elderly (group K, 6 females and 7 males, aged 65 to 75 years [mean age, 72.5 years]), 15 centenarians (group C, 14 females and 1 male, aged 99 to 104 years [mean age, 100.4 years]), and 23 semisupercentenarians (group S, 17 females and 6 males, aged 105 to 109 years [mean age, 106.3 years]). See Table S1 in the supplemental material for further information about the cohort. The study protocol was approved by the Ethics Committee of Sant’Orsola-Malpighi University Hospital (Bologna, Italy) under EM/26/2014/U (with reference to 22/2007/U/Tess).

10.1128/mSystems.00124-20.5 TABLE S1 Summary of age, gender, and medications for each of the 62 subjects and classification in the age group. Download Table S1, XLSX file, 0.01 MB. Copyright © 2020 Rampelli et al. 2020 Rampelli et al. This content is distributed under the terms of the Creative Commons Attribution 4.0 International license.

### Evaluation of the time spent indoors and outdoors by the elderly.

Elderly participants signed the informed consent before undergoing the questionnaires with an interviewer as previously described (37). The participants were asked how often they left their homes (daily, weekly, monthly, etc.) and based on seven different answers were assigned a score: those who never went out, the lowest frequency, were given a score of 1, while those who left their homes “daily,” the highest frequency, were given a score of 7. The answers, treated as a continuous scale (arbitrary scores of 1 to 7), were used to determine the frequency of movement outside home (FMOH) score.

### Library preparation and shotgun sequencing.

DNA libraries were prepared using the QIAseq FX DNA library kit (Qiagen, Hilden, Germany) in accordance with the manufacturer’s instructions. Briefly, total microbial DNA was quantified by a Qubit fluorometer (Invitrogen, Waltham, MA, USA), and 100 ng of each sample was fragmented to a 450-bp size, end-repaired, and A-tailed using FX enzyme mix with the following thermal cycle: 4°C for 1 min, 32°C for 8 min, and 65°C for 30 min. Samples were then incubated at 20°C for 15 min in the presence of DNA ligase and Illumina adapter barcodes for adapter ligation. After two purification steps with Agencourt AMPure XP magnetic beads (Beckman Coulter, Brea, CA, USA), a 10-cycle PCR amplification and a further step of purification as described above, the final library was obtained by pooling the samples at equimolar concentrations of 4 nM. Sequencing was performed on an Illumina NextSeq platform using a 2 × 150-bp paired-end protocol, in accordance with the manufacturer’s instructions (Illumina, San Diego, CA, USA). High-quality paired-end sequences were uploaded to the SRA repository.

### Bioinformatics and biostatistics.

The functional annotation of the sequences deriving from the 62 genomic DNA samples (8) was conducted as previously described (9). In brief, shotgun reads were first filtered by quality and human sequences. This last step was achieved using the human sequence removal pipeline and the WGS read processing procedure of the Human Microbiome Project (HMP) (38). The obtained reads were taxonomically characterized at the species level by MetaPhlAn2 (39) and assigned for functionality at different levels of the KEGG database (40), using Metagenome Composition Vector (MetaCV) with default parameters (41). The resulting table consisted of multiple matrices, with sample identification numbers (IDs) in the columns and annotations at the species level or at different levels of the KEGG database in the rows.

PCoA analysis was carried out using vegan (https://cran.r-project.org/web/packages/vegan/index.html) in R. Significance testing and permutation analysis were performed using the R package stats and vegan. Data separation in the PCoA was tested using a permutation test with pseudo-F ratios (function adonis in the vegan package). When appropriate, *P* values were adjusted for multiple comparisons using the Benjamini-Hochberg correction. A false discovery rate (FDR) of <0.05 was considered statistically significant.

Network plots were determined as previously described (24). In brief, associations between KEGG pathway abundances were evaluated by the Kendall correlation test, displayed with hierarchical Ward linkage clustering based on the Spearman correlation coefficients, and then used to define pathway groups (circles with the same color). Significant associations were verified for multiple testing using the *q* value method (http://www.bioconductor.org/packages/release/bioc/html/qvalue.html) (*P* < 0.05). Permutational multivariate analysis of variance was used to determine whether the pathway groups were significantly different from each other. The network plots were created using Cytoscape software (42). Circle size represents the normalized overabundance of the pathway relative to the background. Connections between nodes represent significant positive Kendall correlations between KEGG pathways (FDR < 0.05).

### Assignment of functions for xenobiotic degradation to commensal bacteria.

Reads with assignment to xenobiotic degradation functions were further inspected for taxonomy. Where present, the species-level classification of MetaCV (41) was retrieved, and the taxon ID in the NCBI taxonomy database was obtained using the web interface of the NCBI Taxonomy Browser tool (https://www.ncbi.nlm.nih.gov/Taxonomy/TaxIdentifier/tax_identifier.cgi). In order to retrieve the entire phylogeny of the assignment, we transformed the NCBI taxonomy IDs into the full lineage by using the ETE3 toolkit (43). Hits for xenobiotic degradation were then split based on their taxonomy and collected in a new table containing the values for each sample. We finally identified the proportion of functions assigned to commensal bacteria of the human core gut microbiome, i.e., microbial taxa that have been found to be shared by all human populations sampled to date (26–30), by specifically looking for their abundance across samples and visualizing them by box plots using the R software.

### Analysis of nutritional data.

Dietary information for the elderly subjects of groups K, C, and S were provided and discussed in our previous publications (1, 8). As regards group Y, the subjects were asked to compile 24-h dietary recalls to retrieve information on the composition of their diet, as previously reported by Barone and colleagues (44). Dietary data for semisupercentenarians (8) were converted to a numeric frequency, in order to infer the daily consumption of each food category. Total daily calorie intake as well as macro- and micronutrient contributions for individuals in groups Y and S were estimated through the MètaDieta software version 3.7 (Meteda, Rome, Italy).

### Data availability.

High-quality paired-end sequences were uploaded to the SRA repository under BioProject number PRJNA553191.
