# Supplementary figures and images for: Shotgun Metagenomics of Gut Microbiota in Humans with up to Extreme Longevity and the Increasing Role of Xenobiotic Degradation
Source: mSystems. 2020 Mar 24;5(2):e00124-20. doi: 10.1128/mSystems.00124-20 (PMC7093822; doi:10.1128/mSystems.00124-20)

A

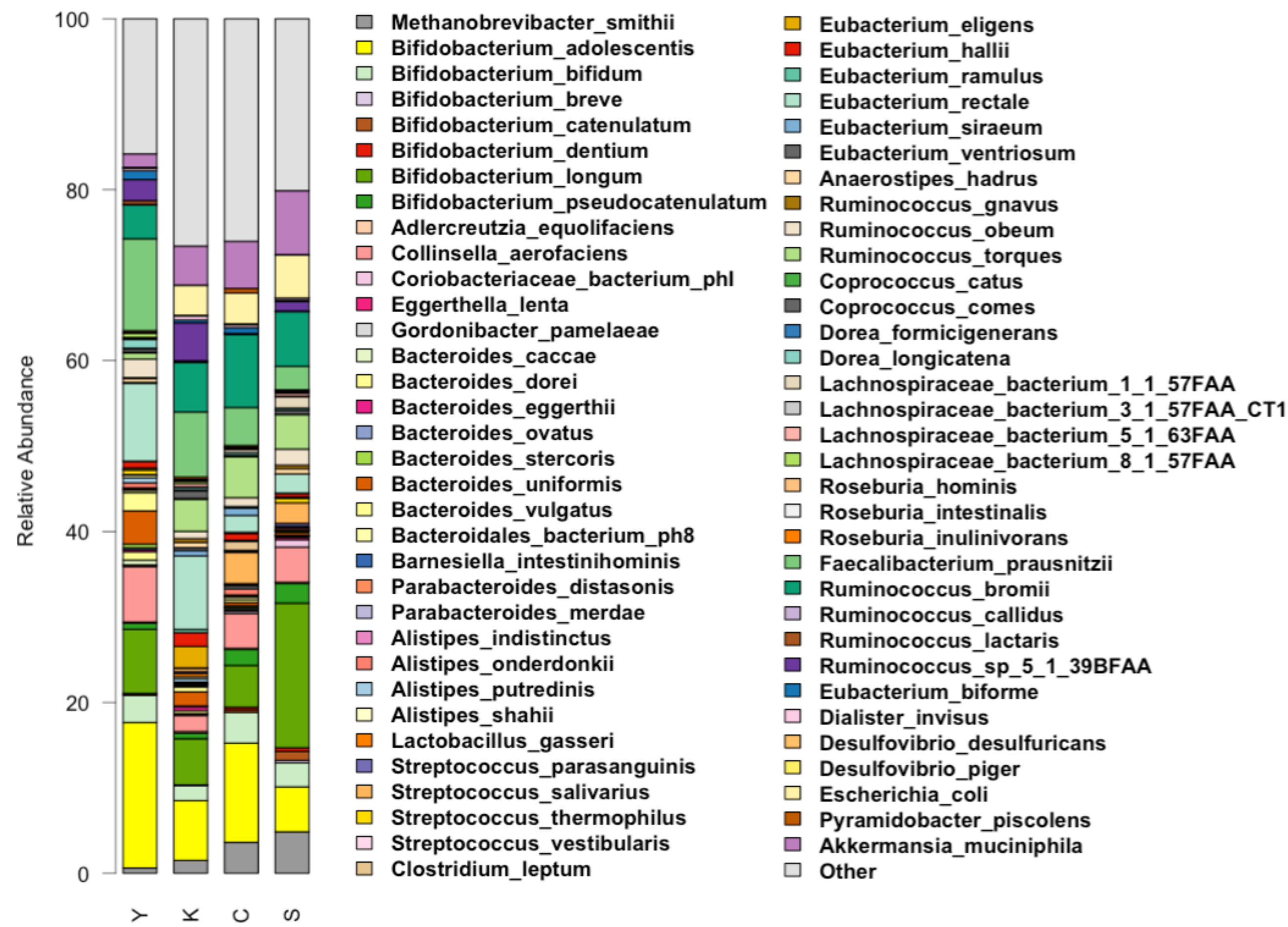

B

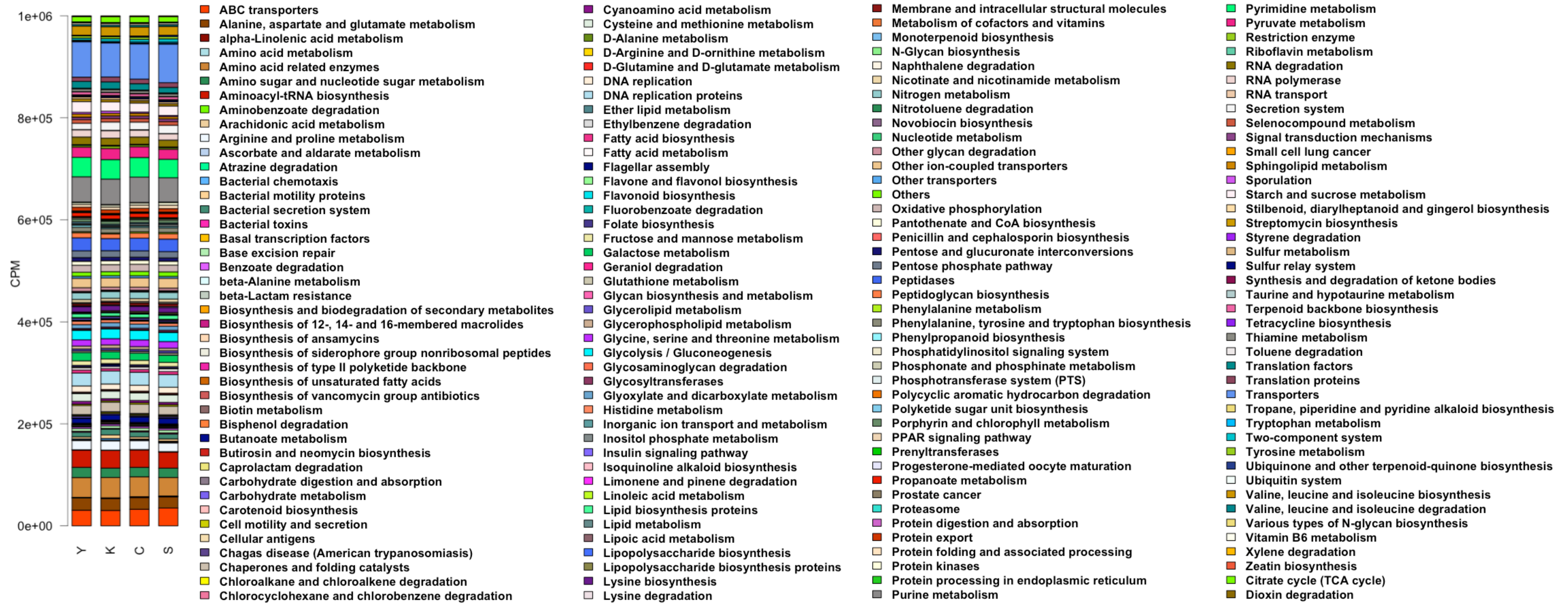

Supplement: FIG S1 [file mSystems.00124-20-sf001.pdf]

## AMINO ACID METABOLISM

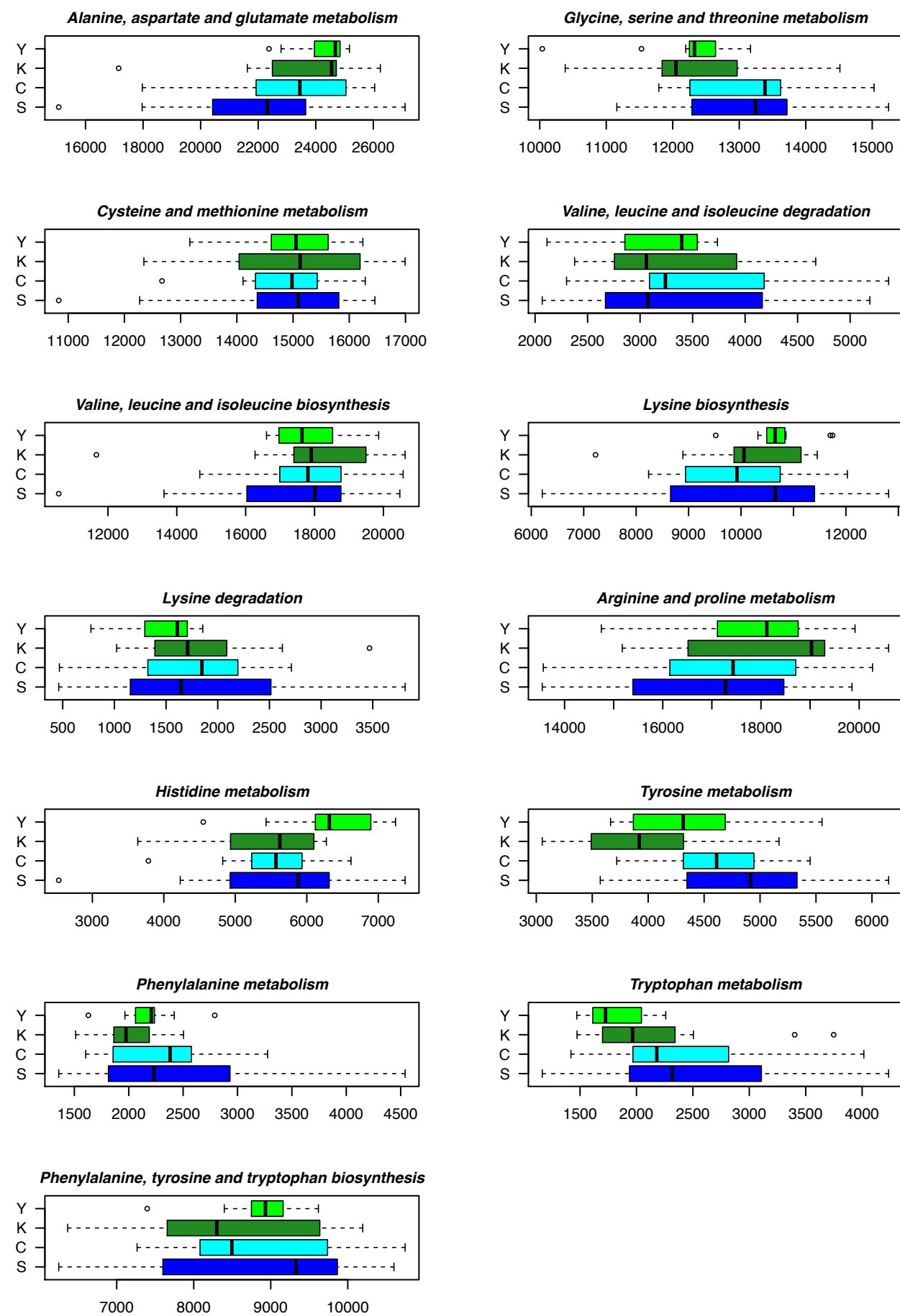

## CARBOHYDRATE METABOLISM

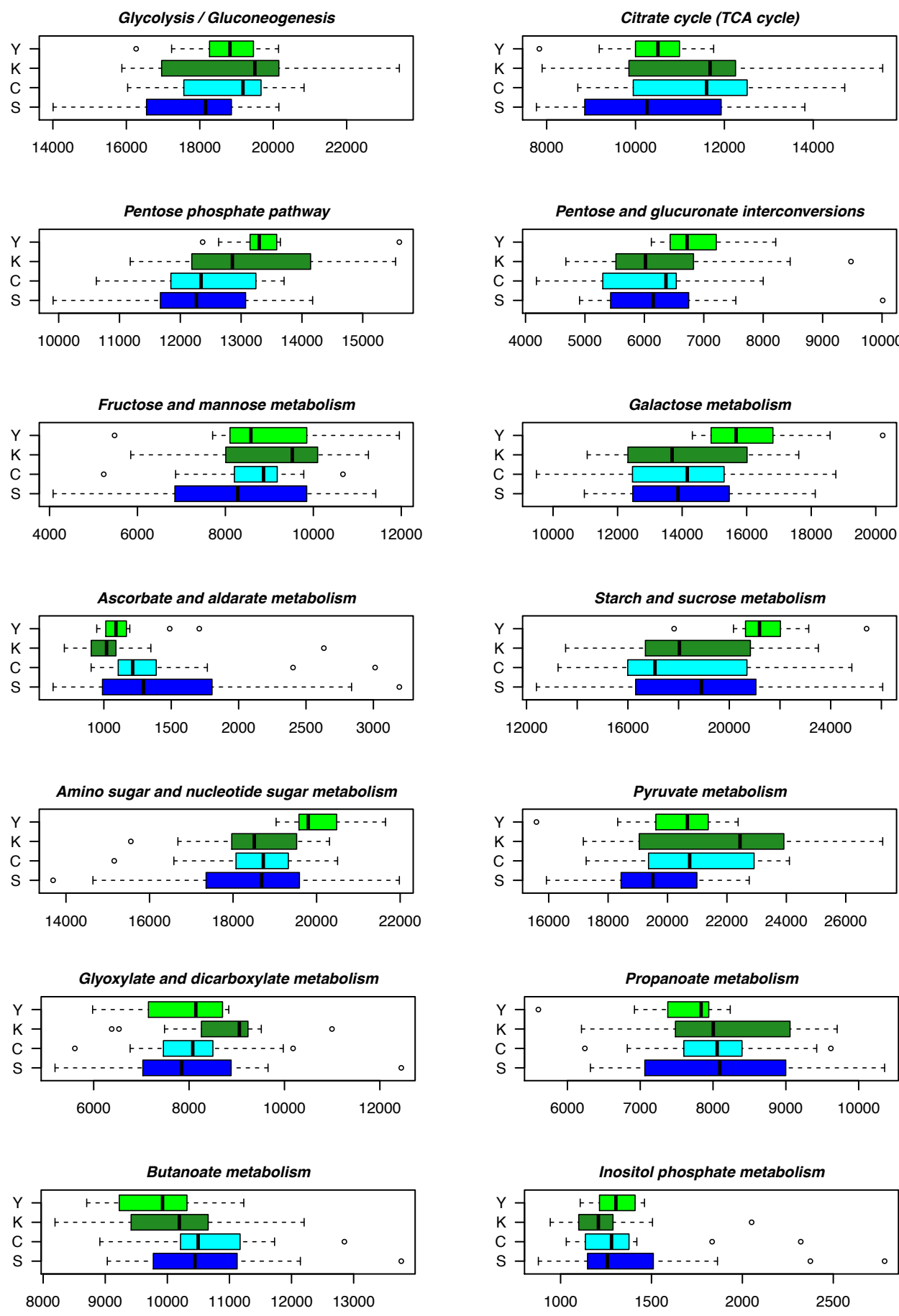

## LIPID METABOLISM

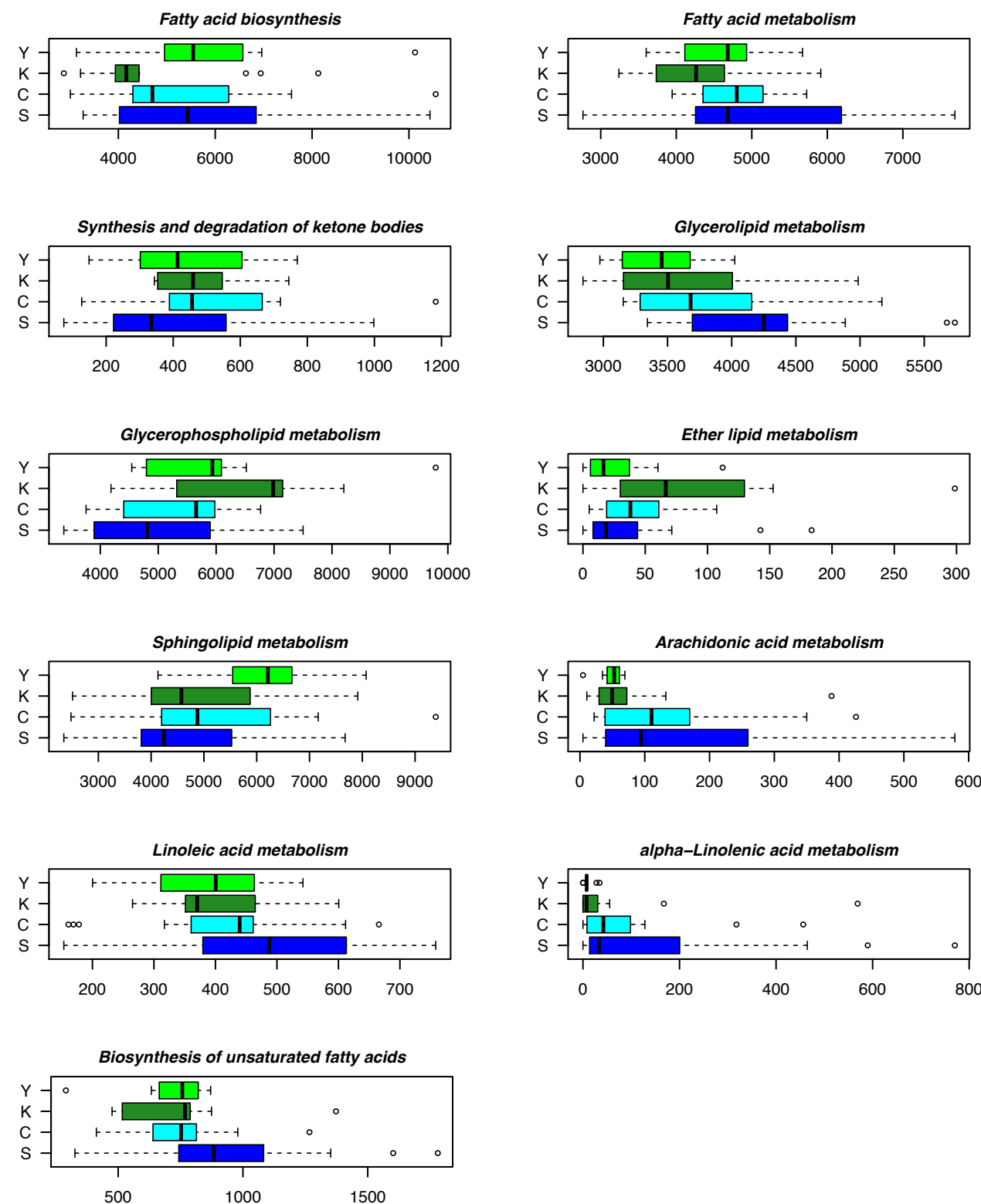

## XENOBIOTIC METABOLISM

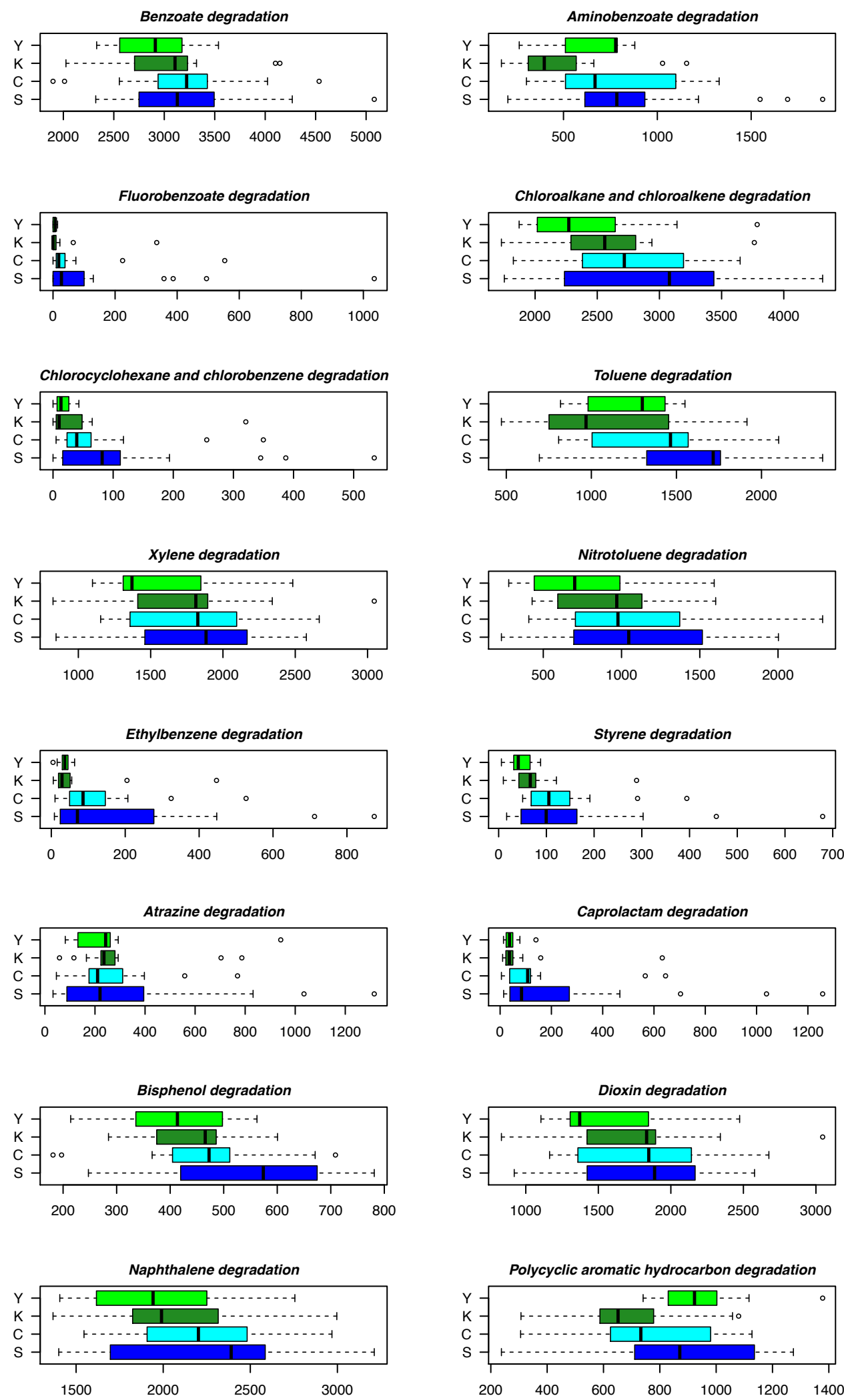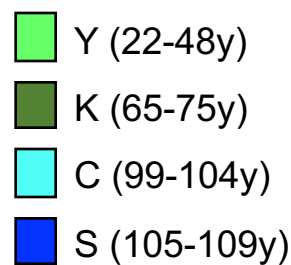

Supplement: FIG S2 [file mSystems.00124-20-sf002.pdf]

# *FMOH score*

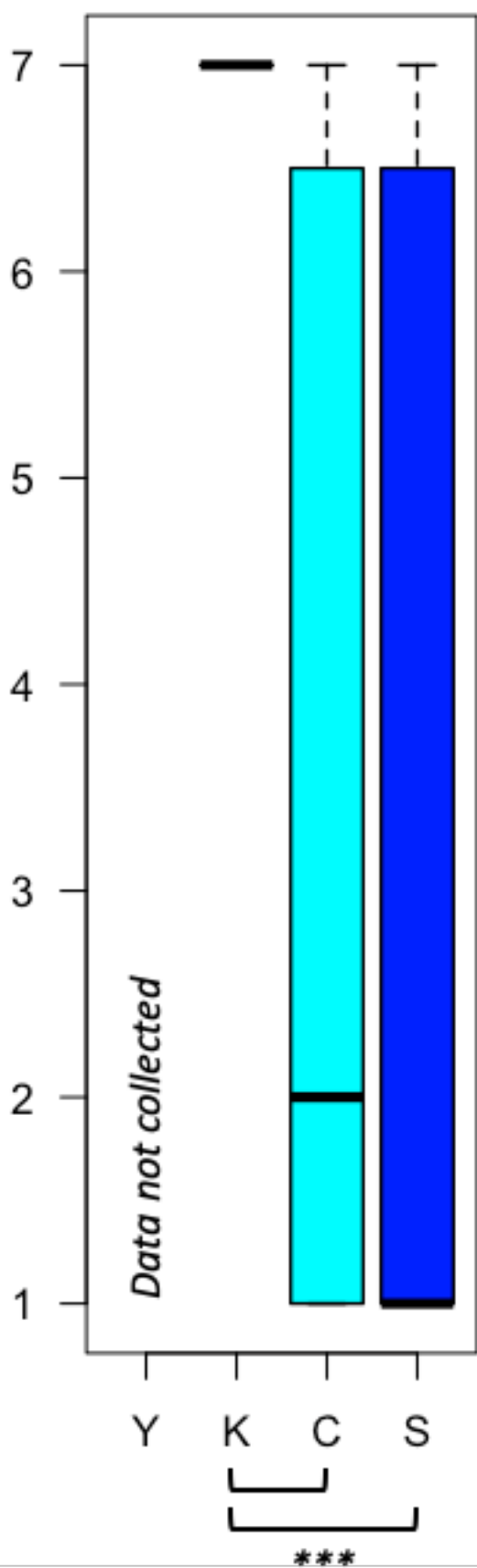

Supplement: FIG S3 [file mSystems.00124-20-sf003.pdf]

Animal fats (g/day) /  
Vegetable fats (g/day)

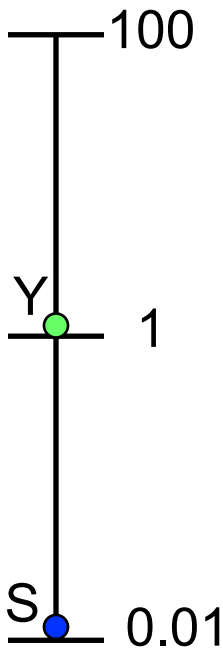

Supplement: FIG S4 [file mSystems.00124-20-sf004.pdf]
